# Iodine Density of Lymphoma, Metastatic SCCA, and Normal Cervical lymph nodes: A Comparative Analysis Based on DLSCT

**DOI:** 10.12688/f1000research.146149.2

**Published:** 2025-01-02

**Authors:** Varalee Mingkwansook, Urusaya Wangprasertkul, Warit Tarathipmon, Arvemas Watcharakorn

**Affiliations:** 1 Radiology, Thammasat University, meung, pathumtani, 12000, Thailand

**Keywords:** Iodine density, contrast-enhanced attenuation value, cervical lymph node, lymphoma, squamous cell cancer, dual-layer spectral computed tomography

## Abstract

**Objective:**

To compare iodine density (ID) and contrast-enhanced attenuation value (CEAV) from dual-layer spectral computed tomography (DLSCT) scans of lymphomatous, metastatic squamous cell carcinoma (SCCA), and normal cervical lymph nodes.

**Methods:**

Data including ID and CEAV were retrospectively collected from patients who underwent DLSCT of the neck between January 2020 and August 2023. Results from each group (lymphomatous, metastatic SCCA, and normal) were compared and analyzed using one-way ANOVA and receiver operating characteristic curve.

**Results:**

129 cervical lymph nodes were collected from patients who met the inclusion criteria (50, 41, and 38 nodes from the lymphomatous, metastatic SCCA, and normal group, respectively). The mean ID of lymphomatous, metastatic SCCA, and normal nodes was 1.01±0.27, 1.36±0.28, and 1.45±0.29 mg/mL, respectively. Comparing lymphomatous nodes with metastatic SCCA nodes, the lymphomatous nodes had significantly lower values of ID (p<0.002) and CEAV (p<0.001). Similarly, when comparing lymphomatous nodes with normal nodes, the lymphomatous nodes had significantly lower values of ID (p<0.001) and CEAV (p<0.001). The optimal ID cut-off value for distinguishing between lymphomatous and metastatic SCCA nodes was 1.175 mg/ml (specificity of 84.2%, sensitivity 77.8%, AUC 0.788, *P* = 0.003). The optimal CEAV cut-off value was 77.5 HU (specificity 88.9%, sensitivity 78.9%, AUC 0.851, *P<*0.001).

**Conclusions:**

The ID and CEAV measurements from DLSCT were significantly different between lymphomatous, metastatic SCCA, and normal lymph nodes. These findings indicate that DLSCT can be used to distinguish between these conditions in the diagnosis of cervical lymph nodes.

## Background

Cervical lymphadenopathy is a common problem, and the malignancy rate of lymph nodes in the neck exceeds 50%. ^1^^–^^5^ The most prevalent etiologies in adults are lymphoma and metastatic squamous cell carcinoma (SCCA). ^2^ These conditions may present with enlarged cervical nodes and upper aerodigestive tract masses which might overlap and be misdiagnosed on a conventional CT ^6^^–^^8^ Dual-layer spectral CT (DLSCT), a subgroup of dual-energy CT (DECT), can generate additional data, including iodine density (ID). ^9^^,^^10^ This study compares ID and contrast-enhanced attenuation value (CEAV) between lymphomatous, metastatic SCCA, and normal cervical lymph node groups.

## Methods

### Study population

The study was approved by the Human Research Ethics Committee of Thammasat University (Medicine) (ref. No. MTU-EC-RA-0-222/66). Patients who underwent DLSCT of the neck with iodinated contrast media between Jan 2020 and Aug 2023 at Thammasat Hospital were retrospectively examined. The inclusion criteria for each group were 1) Metastatic SCCA patients who had primary SCCA of head and neck with pathologically proven metastatic nodes and pre-treatment DLSCT of neck, 2) Lymphoma patients with a final diagnosis of lymphoma involving a neck node with pre-treatment DLSCT of the neck, 3) Normal node patients with DLSCT of the neck for any reason except malignancy, lymphoma, and inflammatory disease. The exclusion criteria were 1) Patients with coexisting primary malignancies (head and neck or another region), and 2) Patients with suboptimal DLSCT, e.g., severe motion or metallic artifact.

### Imaging acquisition

A dual-layer spectral CT scanner, DLSCT (Spectral CT7500 and Philips IQon Spectral CT, Philips Healthcare, Eindhoven, Netherlands), was used to scan all patients using the same standard CT neck protocol of the institute. CT parameters were as follows: 1.4:1 helical pitch, 0.4-second rotation time, and 120 kVp tube voltage with a reconstructed FOV of 512 mm2. Automated mAS and beam collimation were registered and differed depending on body habitus. The detector configurations were 128×0.625 mm for Spectral CT7500 and 64×0.625 mm for Philips IQon Spectral CT.

CT neck was performed along the skull base to the aortic arch in the craniocaudal fashion. Intravenous injection of non-ionic iodinated contrast agent (Ultravist 300 mg/ml, Bayer Vital GmbH, Leverkusen, Germany, catalogue numbers: 064-18-27492-00) with 30-mL saline chaser (0.9% sodium chloride, Thai Nakorn Patana, Nonthaburi, Thailand, catalogue numbers: F-013-13035) was administered at the rate of 2.5 mL/sec using a dual syringe injection system (Stellant, MEDRAD, Indianola, Pennsylvania). The non-ionic iodinated contrast agent dosage varied according to body weight (1.5 mL/kg). Enhanced images were obtained 70 seconds after contrast agent injection.

### Imaging post-processing

**Figure 1.**
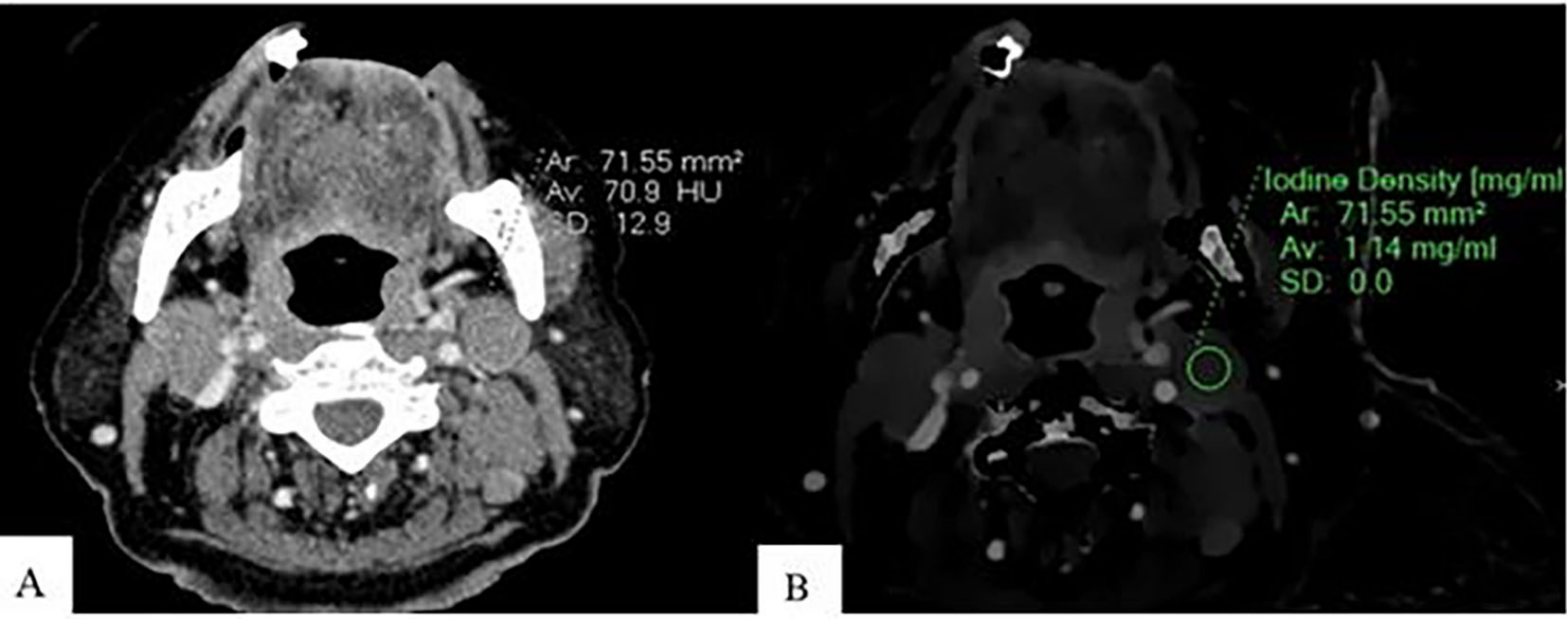
Measurement of ID and CEAV in lymphomatous node. An Axial CT image of a patient with lymphoma shows CEAV measurement (70.9 HU) of the enlarged lymph node at left cervical level IIA (A). An ID measurement (1.14 mg/ml) of this lymph node at the same CT level (B).

**Figure 2.**
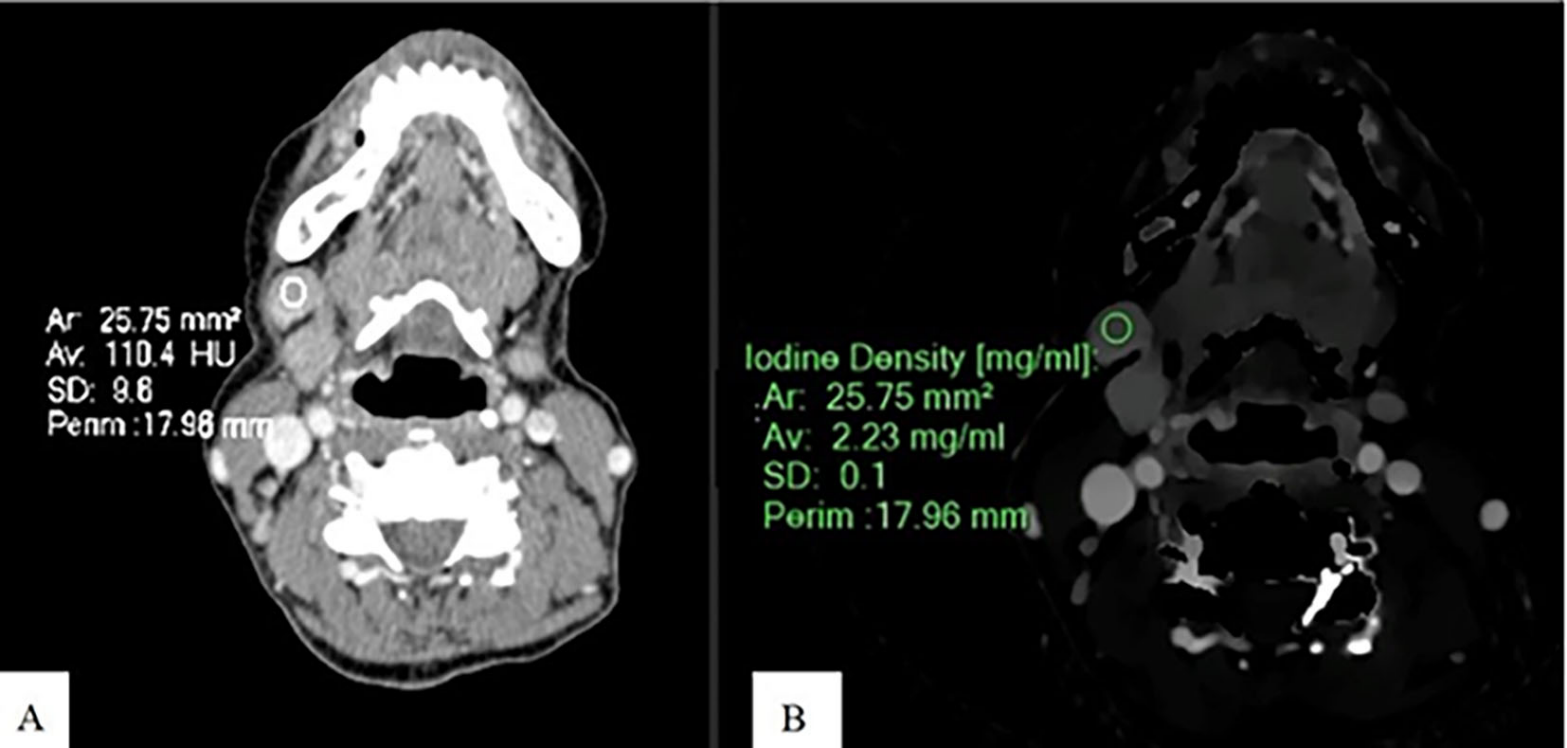
Measurement of ID and CEAV in metastatic SCCA node. An Axial CT image of a patient with SCCA at gum shows CEAV measurement (110.4 HU) of the enlarged lymph node at left cervical level IB (A). An ID measurement (2.23 mg/ml) of this lymph node at the same CT level (B).

**Figure 3.**
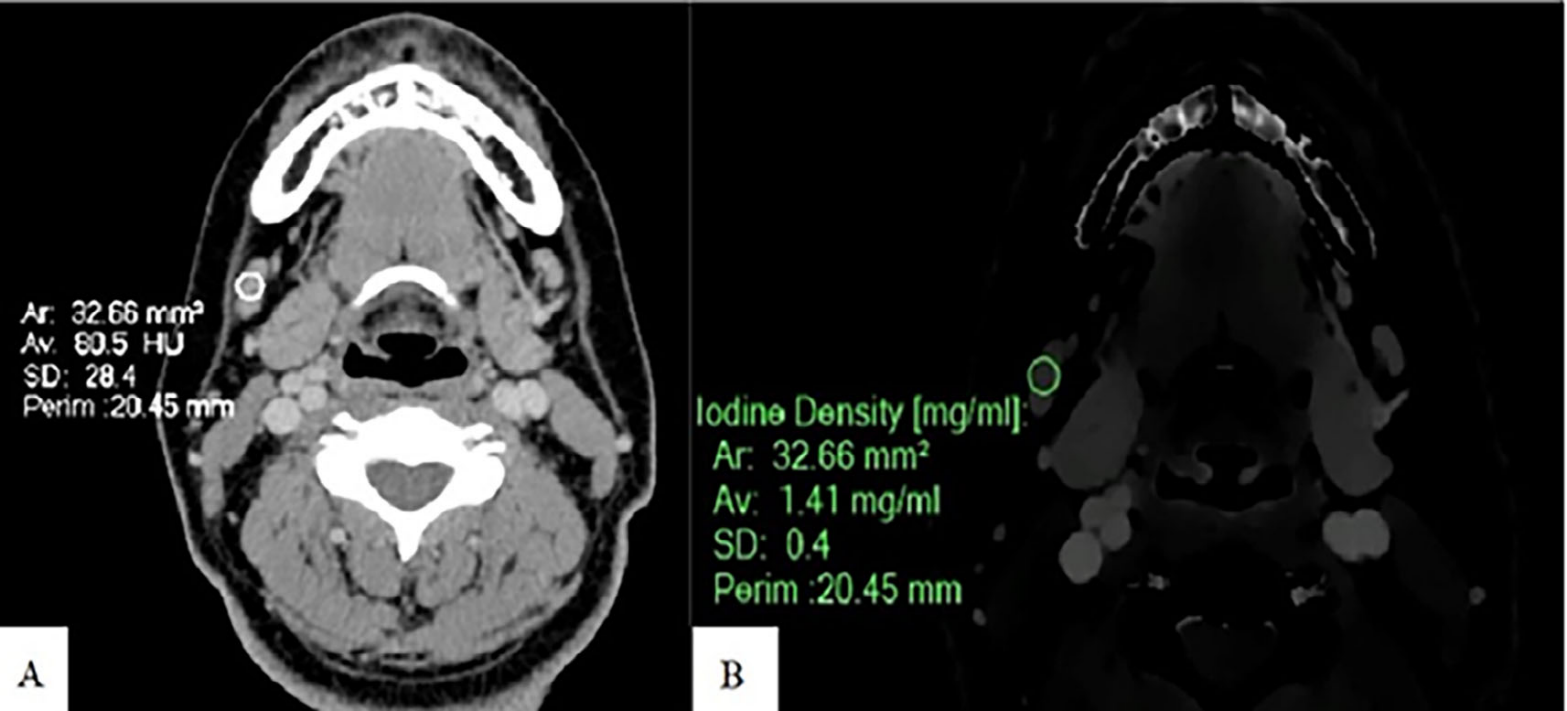
Measurement of ID and CEAV in normal node. An Axial CT image of a patient with normal lymph nodes shows CEAV measurement (80.5 HU) of a right cervical level IB node (A). An ID measurement (1.41 mg/ml) of this lymph node at the same CT level (B).

Spectral post-processing workstation (IntelliSpace Portal v11, Philips Healthcare, Netherlands) was used togenerateID and contrast-enhanced images ( Figures 1, 2, and 3).

### Image assessment

ID and CEAV were measured by placing rounded or oval-shaped regions of interest (ROI) on axial images in which lymph nodes appeared the largest ( Figures 1, 2, and 3). Regions of interest (ROIs) for each lymph node were delineated to encompass the largest possible area within the solid portion of the node, ensuring that each ROI had a minimum size of 6 mm ^2^. Areas of necrosis, cysts, calcifications, and fat were systematically excluded from the ROI placement. In instances where the solid portion exhibited heterogeneous enhancement, the largest feasible ROI was selected to ensure accurate and reliable measurements. The mean Hounsfield unit (HU) of each ROI was used as the representative value for the lymph node. ROIs were placed on axial images where the lymph nodes displayed the most prominent enhancement, with a maximum of three nodes selected for analysis. When the lymph node showed consistent enhancement across multiple slices, the slice displaying the largest cross-sectional area was chosen to obtain the most representative ROI. All lymph nodes were evaluated by a radiologist with 15 years of experience in head and neck imaging. The average ID and CEAV values of each patient were recorded.

The selected lymphomatous and metastatic SCCA nodes had to be enlarged (>8 mm in the short axis) or have other radiologic characteristics of pathologic nodes.

To achieve reliable ROI, normal nodes had to be <8 mm and >5 mm in the short axis without other radiologic signs of pathologic nodes.

ID and CEAV of the sternocleidomastoid muscle (SCM) and internal jugular vein (IJV) were obtained from every patient using circular or ellipsoid ROIs.

### Standard of reference

Lymphomatous nodes were selected from patients with histopathologically proven lymphoma from cervical nodes or other head and neck areas. The selected lymphomatous nodes showed evidence of post-treatment regression on follow-up imaging. Metastatic SCCA nodes selected on CT had to correlate with the operative report and pathological results regarding side, nodal station, and size. Normal cervical lymph nodes were included in patients who were free from malignancy or inflammation in the neck.

### Statistics

ID and CEAV were interpreted in terms of mean, standard deviation (SD), median, and interquartile range (IQR). One-way ANOVA was used to compare mean ID and CEAV between the 3 groups. ROC curve was used to estimate the area under the curve (AUC) with 95%CI for differential diagnosis between lymphomatous and metastatic SCCA nodes. Descriptive statistics was used to evaluate lymph node diameter.

## Results

### Study population

There was a total of 129 cervical nodes from 55 patients. The number of lymphomatous, metastatic SCCA, and normal cervical nodes was 50, 41, and 38, respectively. Baseline demographic, lymph node diameter, level of lymph nodes, and site of primary lesion of SCCA, including histopathologic subtypes of lymphoma, are described in Table 1.

**Table 1.** Clinical data of lymphomatous, metastatic SCCA and normal groups.

	Lymphoma	Metastatic SCCA	Normal
Age (Mean±SD)	59.10±16.95	61.59±13.89	48.50±17.40
Gender			
	9 female	3 female	10 female
	11 male	14 male	8 male
Number of lymph nodes	50	41	38
Level of lymph nodes	1a (n=2) 1b (n=8) 2a (n=14) 2b (n=3) 3 (n=10) 4 (n=10) 5a (n=3) 5b (n=0) 6 (n=0)	1a (n=2) 1b (n=7) 2a (n=21) 2b (n=7) 3 (n=2) 4 (n=1) 5a (n=0) 5b (n=0) 6 (n=1)	1a (n=1) 1b (n=13) 2a (n=19) 2b (n=2) 3 (n=2) 4 (n=0) 5a (n=1) 5b (n=0) 6 (n=0)
Average diameter of lymph nodes	14.16 (10.15-17.63)	10.64 (9.55-19.10)	5.82 (5.41-6.32)
Note	Histologic subtypes - Hodgkin lymphoma (n=6) - Non Hodgkin lymphoma (n=38) - Diffuse large B cell (n=10) - Follicular cell (n=12) - NLPHL (n=2) - T cell (n=8) - Marginal zone (n=3) - Round cell (n=3)	Site of primary CA - Nasopharynx (n=11) - Nasal septum (n=2) - Tongue (n=3) - Floor of mouth (n=6) - Gum (n=5) - Retromolar trigone (n=2) - Tonsil (n=2) - Pyriform sinus (n=3) - Supraglottis (n=7)	-

### Iodine density (ID) and contrast-enhanced attenuation value (CEAV)

**Figure 4.**
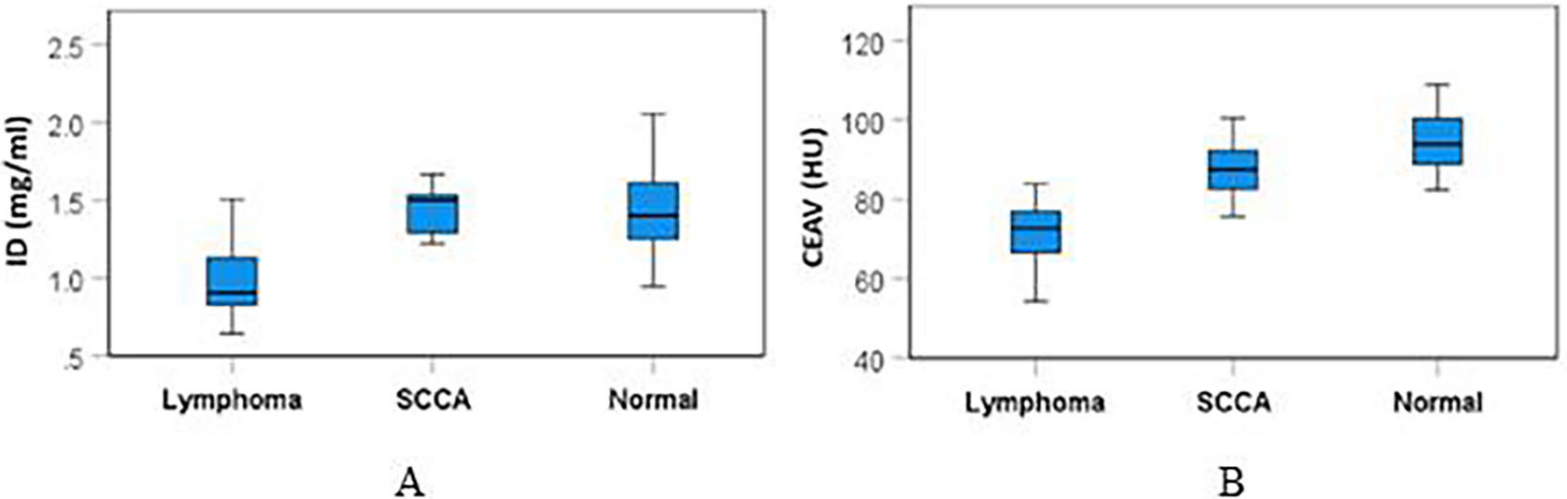
Box and whiskers plots of ID (A) & CEAV (B) of lymphoma, metastatic SCCA and normal nodes.

The mean (±SD) ID and CEAV of lymphomatous, metastatic SCCA, and normal cervical nodes are summarized in Table 2 and Figure 4. There were significant differences of ID (p<0.001) and CEAV (p<0.001) between the three groups.

**Table 2.** Comparison of parameters between lymphomatous, metastatic SCCA and normal nodes.

Patient group	Lymphoma	SCCA	Normal	p-value
ID of lymph nodes (mg/ml)	1.01±0.27	1.36±0.28	1.45±0.29	<0.001 *
ID of SCM (mg/ml)	0.45±0.19	0.44±0.24	0.42±0.18	0.928
ID of IJV (mg/ml)	3.67±0.76	3.30±0.62	3.25±0.72	0.140
CEAV of lymph nodes (HU)	72.91±10.69	86.84±8.95	95.90±9.67	<0.001 *
CEAV of SCM (HU)	68.27±7.31	67.27±9.74	68.27±5.51	0.848
CEAV of IJV (HU)	150.94±23.56	144.38±19.61	149.58±20.10	0.635

Results show Mean±SD.

* Statistically significant at p-value<0.05 determined by One-way ANOVA.

Subgroup analysis showed that the ID of lymphomatous nodes (1.01±0.27 mg/ml) was significantly lower than the ID of metastatic SCCA (1.36±0.28 mg/ml), p=0.002, and it was also lower than the ID of normal nodes (1.45±0.29 mg/ml), p<0.0001.

CEAV of lymphomatous nodes (72.91±10.69 HU) was significantly lower than the CEAV of metastatic SCCA (86.84±8.95 HU), p<0.0001, and it was lower than the CEAV of normal nodes (95.90±9.67 HU), p<0.0001. Comparative results of ID and CEAV between subgroups are summarized in Table 3.

**Table 3.** Pairwise comparison of iodine density and contrast enhanced attenuation value between each group.

	Mean difference	Std. Error	p-value
**ID**			
Normal – Lymphoma	0.44	0.09	<0.001 *
Normal – SCCA	0.09	0.09	0.640
Lymphoma – SCCA	-0.35	0.09	0.002 *
**CEAV**			
Normal – Lymphoma	22.98	3.20	<0.001 *
Normal – SCCA	9.06	3.33	0.032 *
Lymphoma – SCCA	-13.93	3.25	<0.001 *

* Statistically significant at p-value<0.05 determined by post-hoc test (Scheffe).

There was no significant difference in ID or CEAV of the internal jugular vein (IJV) or sternocleidomastoid muscles (SCM) between the three groups, p≥0.05 ( Table 2).

### Receiver operating characteristic curve

**Figure 5.**
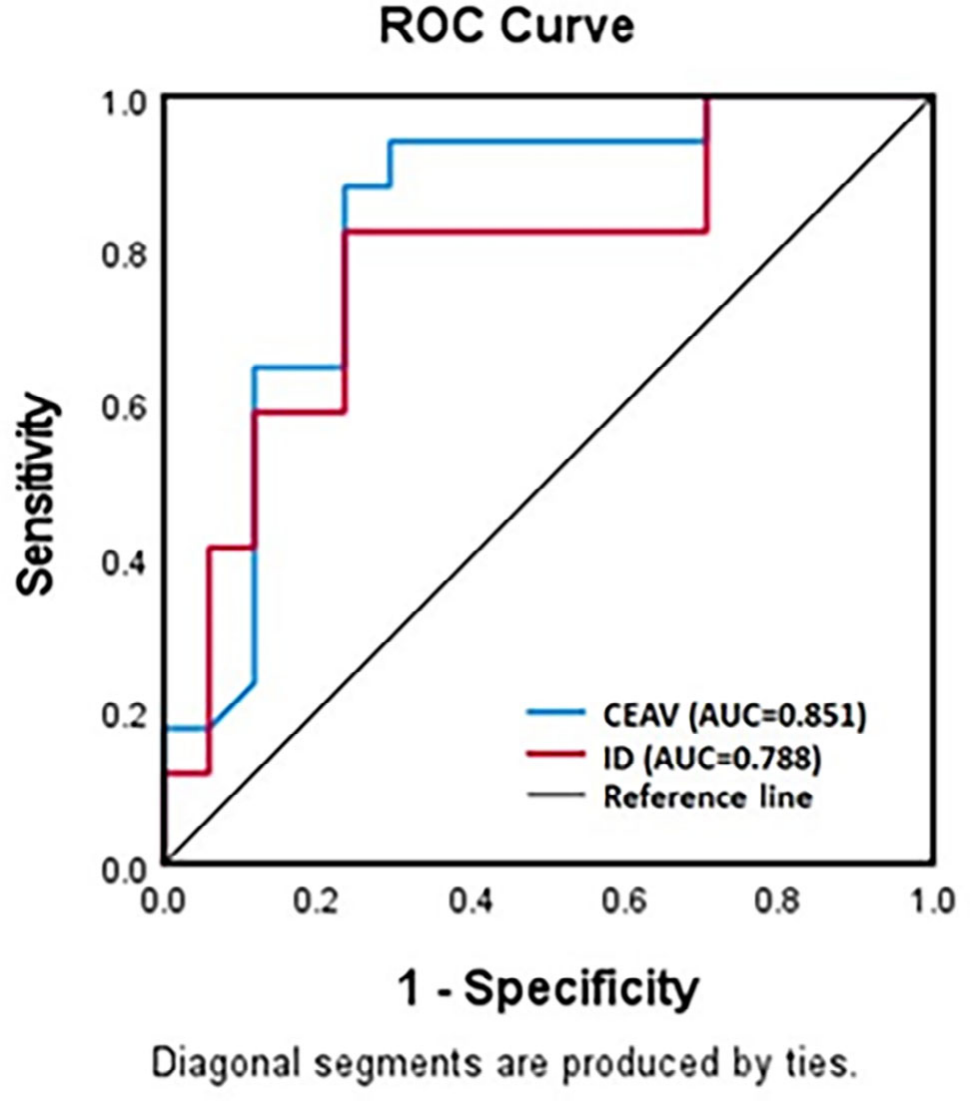
ROC curves for ID and CEAV as test to diagnose lymphomatous or metastatic SCCA nodes. Both curves show high diagnostic performance.

The ROC curve for ID and CEAV in differentiation between lymphoma and SCCA showed good diagnostic performance with the area under the curve at 0.788 (95% CI=0.632-0.944) and 0.851 (95% CI=0.721-0.982), respectively ( Figure 5).

The optimal cut-off value of ID to differentiate lymphomatous nodes from metastatic SCCA, as derived from the ROC curve, was 1.175 mg/ml with a sensitivity of 85% and specificity of 87.5 %. The CEAV at 77.5 HU could also differentiate between these entities, with a specificity of 88.9% and a sensitivity of 78.9%.

## Discussion

This is the first study to use DLSCT to compare ID between lymphomatous, metastatic SCCA, and normal cervical lymph nodes. We found lymphomatous nodes had significantly lower ID and CEAV than metastatic SCCA and normal nodes. Our data suggested that a cut-off value of ID at 1.175 mg/ml may enable discrimination between lymphomatous and SCCA nodes with specificity of 84.2% and sensitivity of 77.8%.

Some literature reported ID of cervical nodes studied on dual-sources or rapid-switch technique DECT. Prior studies rated ID and CEAV highest in normal nodes, followed by metastatic and then lymphomatous nodes, similar to our findings. ^2^^,^^11^^,^^12^ A study by Yang et al. also found lower ID of lymphomatous nodes than of metastatic SCCA nodes, but they did not show a statistical difference between these two diseases. ^2^ They performed the study using a rapid switch kVp machine (Gemstone spectral imaging) with acquisition time at 30 seconds after contrast administration, which might explain why their results differ from ours.

The results from this study differ from results of previous studies performed in the abdominal area, which showed higher ID in lymphomatous nodes than in metastatic nodes. ^13^ Many studies demonstrated different IDs among various types of tumors. ^14^^–^^16^ Differences of histopathological types and vascular supply between neck and abdominal structures could explain these different ID results.

Our results showed a lower ID of metastatic SCCA nodes than of normal neck nodes, which was similar to prior studies. ^17^^–^^19^ This might relate to HU distribution, which is highest at the center of normal nodes, whereas it is evenly distributed in metastatic nodes. ^20^ As normal nodes must be less than 8 mm in diameter, the ROI for ID measurement was inevitably placed at their center, resulting in a relatively higher ID. Greater vessel density in normal nodes than in metastatic lymph nodes with possible micronecrosis might explain this result. ^19^^,^^21^

The ID of metastatic SCCA nodes in this study (1.36±0.28 mg/ml) was lower than the ID in a study by Tawfik et al. (2.34±0.45 mg/ml) but higher than in the studies reported by Foust et al. (0.96±0.28 mg/ml) and Luo et al. (0.98±0.42 mg/ml). ^17^^,^^19^^,^^22^ Their studies were performed by dual-source DECT (Somatom; Siemens Healthcare, Erlangen, Germany) with different iodine concentrations of contrast media (350-400 iodine/ml). These could result in different ID values of the cervical lymph nodes with the same pathologic type.

This study also showed a relatively lower ID in normal nodes (1.45±0.29 mg/ml) compared to a prior result from Sauter AP et al. (2.09±0.09 mg/ml), which also used a DLSCT machine (iQon, Philips Healthcare, Cleveland, OH, USA). ^23^ In contrast to this study, they used a higher iodine concentration of contrast media (Imeron 400) with a larger contrast volume (150 ml) on their CT neck protocol. These resulted in higher total iodine load and could have caused the higher ID values in their study.

We also compared the CEAV of lymph nodes between each group. This approach is more practical for routine interpretation because it can be directly measured on PACS. The results corresponded to the ID results, with lowest values in lymphomatous, metastatic SCCA, and normal nodes, respectively. CEAV was notably significantly different between the three groups (p-value<0.001).

As bolus tracking was not included in our routine CT neck protocol, we measured ID and CEAV of the sternocleidomastoid muscle (SCM) and internal jugular vein (IJV) to compare iodine content among the three groups. The results showed no significant difference in ID and CEAV of these two structures between the three patient groups (p-value>0.05).

This study has several limitations. First, it was conducted retrospectively with small patient groups. Iodine qualification data were unavailable for some patients because they were referred to our health care center after having undergone conventional CT. Second, we did not compare ID and CEAV between each lymph node level because some nodes were too small, and we did not have nodes in levels III-VI in the normal group. Comparison between normal and diseased nodes in these cervical levels might affect the results. A larger-scale study including each cervical level might address this limitation. Third, considering prior studies which showed different ID between different types of tumors, ^14^^–^^16^ we hypothesized that ID might be affected by the histologic subtype of lymphoma. Study of ID in various lymphomatous subtypes might improve subtype differentiation. Fourth, not all lymph nodes in the lymphomatous group were confirmed because lymph node dissection was not standard treatment for this disease. Fifth, the ROI placement in this study was performed by a single reader, which precludes the evaluation of inter-reader variability. However, efforts were made to standardize the process and ensure consistency throughout the analysis. Incorporating multiple readers in future studies could provide additional insights into the reproducibility and reliability of the findings.

## Conclusion

The results of our study indicate that ID and CEAV findings from DLSCT differed significantly between lymphomatous, metastatic SCCA, and normal cervical nodes. This knowledge improves differentiation of these conditions.

### Statements and declarations

#### Ethical approval and consent to participate

The Human Research Ethics Committee of Thammasat Univerity (Medicine) approved conducting this research with the certificate project number MTU-EC-RA-0-222/66, and approved on December 20, 2023. The inform-consent was waived requirement due to the retrospective nature of the study.

## Author contributions

MV made contributions in literatures search, study design, data interpretation, draft writing, critical revision and final approval of the final version for submitted. WU made contributions in literatures search, data collection, data analysis and interpretation, and TW and WA made contributions in draft writing, critical revision and also faculty collaborations.

## Data Availability

Zenodo: Iodine density of lymphoma, metastatic SCCA, and normal cervical lymph nodes: Based on DLSCT. https://doi.org/10.5281/zenodo.11120918. ^24^ This project contains the following underlying data: Patients demographic data, iodine density and contrast attenuation value of neck nodes for Final data_18 Mar 2024_Final.xlsx. Data are available under the terms of the Creative Commons Attribution 4.0 International license (CC-BY 4.0). ***Reporting guidlines*** Zenodo: STARD checklist. https://doi.org/10.5281/zenodo.11120918. ^24^
