# Supplementary material for: Iodine Density of Lymphoma, Metastatic SCCA, and Normal Cervical lymph nodes: A Comparative Analysis Based on DLSCT
Source: F1000Res. 2025 Jan 2;13:498. Originally published 2024 May 17. [Version 2] doi: 10.12688/f1000research.146149.2 (PMC11739862; doi:10.12688/f1000research.146149.2)
Supplement: Peer review history

# Reviewer report: Reviewer response for version 2

**DOI:** 10.5256/f1000research.176267.r355399

**Published:** 2025-01-15

**Authors:** Siriwan Piyapittayanan

**Affiliations:** 1 Faculty of Medicine, Siriraj Hospital, Mahidol University, Bangkok, Thailand

No new commment

Is the work clearly and accurately presented and does it cite the current literature?

Yes

If applicable, is the statistical analysis and its interpretation appropriate?

Yes

Are all the source data underlying the results available to ensure full reproducibility?

Yes

Is the study design appropriate and is the work technically sound?

Yes

Are the conclusions drawn adequately supported by the results?

Yes

Are sufficient details of methods and analysis provided to allow replication by others?

Yes

Reviewer Expertise:

Neuroradiology

I confirm that I have read this submission and believe that I have an appropriate level of expertise to confirm that it is of an acceptable scientific standard.

# Reviewer report: Reviewer response for version 2

**DOI:** 10.5256/f1000research.176267.r355400

**Published:** 2025-01-03

**Authors:** Jarunee Intrapiromkul

**Affiliations:** 1 Neuroradiology Radiology, Nuclear Medicine, Pediatric Radiology, West Virginia University, Johns Hopkins University, Morgantown, Baltimore, USA

No additional comment.

Is the work clearly and accurately presented and does it cite the current literature?

Yes

If applicable, is the statistical analysis and its interpretation appropriate?

Yes

Are all the source data underlying the results available to ensure full reproducibility?

Partly

Is the study design appropriate and is the work technically sound?

Partly

Are the conclusions drawn adequately supported by the results?

Yes

Are sufficient details of methods and analysis provided to allow replication by others?

Partly

Reviewer Expertise:

Head and neck imaging, perfusion imaging

I confirm that I have read this submission and believe that I have an appropriate level of expertise to confirm that it is of an acceptable scientific standard.

# Reviewer report: Reviewer response for version 1

**DOI:** 10.5256/f1000research.160196.r344815

**Published:** 2024-12-20

**Authors:** Siriwan Piyapittayanan

**Affiliations:** 1 Faculty of Medicine, Siriraj Hospital, Mahidol University, Bangkok, Thailand

Comments

 1. Study design; generally, metastatic cervical lymph nodes are not only SCCA. This study has not include another cell type of metastatic lymph node such as thyroid carcinoma, salivary gland carcinoma or metastatic node from organ outside head and neck such as breast and lung cancer. Explain the reason that why this study include only SCCA nodal metastasis.  

 2. Heading; may put some word in the heading such as comparative, can iodine density differentiate between, to increasingly interest.

Is the work clearly and accurately presented and does it cite the current literature?

Yes

If applicable, is the statistical analysis and its interpretation appropriate?

Yes

Are all the source data underlying the results available to ensure full reproducibility?

Yes

Is the study design appropriate and is the work technically sound?

Yes

Are the conclusions drawn adequately supported by the results?

Yes

Are sufficient details of methods and analysis provided to allow replication by others?

Yes

Reviewer Expertise:

Neuroradiology

I confirm that I have read this submission and believe that I have an appropriate level of expertise to confirm that it is of an acceptable scientific standard.

# Response

**Published:** 2024-12-21

**Authors:** Varalee Mingkwansook

**Affiliations:** Radiology, Thammasat University, Pathum Thani, Pathum Thani, Thailand

Thank you for your valuable comment.

 1. The decision to focus solely on metastatic SCCA in cervical lymph nodes was made to ensure a more controlled and focused analysis. SCCA is one of the most common causes of metastatic cervical lymphadenopathy, particularly in head and neck cancers. By concentrating on SCCA, we aimed to investigate its specific iodine density patterns in relation to lymphoma. Including other types of metastatic lymph nodes, such as those from thyroid carcinoma, salivary gland carcinoma, or cancers from distant organs like the breast or lung, would introduce significant variability due to differences in tumor biology, vascularity, and iodine uptake. This variability could complicate the analysis and make it difficult to draw clear comparisons between the two malignancies. Focusing on SCCA allowed for a more homogenous study sample, providing clearer insights into iodine density patterns specific to SCCA and lymphoma, which is the primary goal of this research. We plan to explore a broader comparison in future studies.

 2. The heading will be modified to "Iodine Density of Lymphoma, Metastatic SCCA, and Normal Cervical Lymph Nodes: A Comparative Analysis Using DLSCT"

# Reviewer report: Reviewer response for version 1

**DOI:** 10.5256/f1000research.160196.r282853

**Published:** 2024-08-08

**Authors:** Jarunee Intrapiromkul

**Affiliations:** 1 Neuroradiology Radiology, Nuclear Medicine, Pediatric Radiology, West Virginia University, Johns Hopkins University, Morgantown, Baltimore, USA

For ROI measurement, there is no clear description on how to place the ROI in the lymph nodes. Is the size of ROI the same in all cases? Is it placed on the solid portion? For lymph nodes with heterogeneous enhancement, do you place it on the most enhanced portion or cover all lymph nodes. The details of ROI placement should be described in more detail so that the study can be reproduce. Also, having a second reader placing the ROI is a great way to ensure the study reproducibility by evaluating the inter-reader variability. The mean value from 2 readers can then be used for analysis.

 The proposed cut-off ID value may depend on the contrast concentration and dosage, which are varied on each imaging centers.

Is the work clearly and accurately presented and does it cite the current literature?

Yes

If applicable, is the statistical analysis and its interpretation appropriate?

Yes

Are all the source data underlying the results available to ensure full reproducibility?

Partly

Is the study design appropriate and is the work technically sound?

Partly

Are the conclusions drawn adequately supported by the results?

Yes

Are sufficient details of methods and analysis provided to allow replication by others?

Partly

Reviewer Expertise:

Head and neck imaging, perfusion imaging

I confirm that I have read this submission and believe that I have an appropriate level of expertise to confirm that it is of an acceptable scientific standard, however I have significant reservations, as outlined above.

# Response

**Published:** 2024-08-22

**Authors:** Varalee Mingkwansook

**Affiliations:** Radiology, Thammasat University, Pathum Thani, Pathum Thani, Thailand

**For ROI measurement, there is no clear description on how to place the ROI in the lymph nodes.**

**- Is the size of ROI the same in all cases?**

 - ROI placement for each lymph node was drawn in the largest area as much as possible in the solid portion. The size must be at least 6 mm ^2^.

**- Is it placed on the solid portion?**

             - Yes. ROIs were placed on the solid enhancing portion. Areas of necrosis, cyst, calcification or fat were carefully avoided.

**- For lymph nodes with heterogeneous enhancement, do you place it on the most enhanced portion or cover all lymph nodes.**

            - If the solid portion (excluding necrosis, cystic area, fat and calcification) was heterogenously enhanced, we drawn the largest ROI as much as possible to acheive the reliable ROI. The mean HU of each ROI was used as a representative for each lymph node.

**- The details of ROI placement should be described in more detail so that the study can be reproduce.**

             - ROIs were placed on axial images in which lymph nodes appeared the most prominent enhancement (3 nodes maximum). If the lymph node had similar degree of enhancement on each axial view, the slice which lymph node appeared to be largest was selected in order to achieve the largest ROI. ROI must be at least 6 mm ^2^ in size. We drew largest ROI for each lymph node as much as possible, avoiding areas of necrosis, cyst, calcification or fat.

**- Also, having a second reader placing the ROI is a great way to ensure the study reproducibility by evaluating the inter-reader variability. The mean value from 2 readers can then be used for analysis.**

             - We will conduct the new article with more subjects and providing inter-reader variability in the future.

**- The proposed cut-off ID value may depend on the contrast concentration and dosage, which are varied on each imaging centers**

             - The authors agree with this issue which already mentioned in discussion section.
